# Language skills of adults with dyslexia in English as a foreign language: proposal of a language spontaneity deficit hypothesis

**DOI:** 10.1007/s11881-025-00326-1

**Published:** 2025-03-26

**Authors:** Uxue Pérez-Litago, Josué M. Rojas-Guerra, Cristina Martínez-García, Paz Suárez-Coalla

**Affiliations:** Department of Psychology, University of Oviedo, Oviedo, Spain

**Keywords:** Adults, Dyslexia, Foreign language, Oral production, Reading comprehension, Writing production

## Abstract

Developmental dyslexia is characterized by reading and writing deficits that persist into adulthood. However, the mechanisms underlying these deficits appear to affect several language domains negatively. The present study aims to investigate how 18 native Spanish-speaking adults with developmental dyslexia perform different language tasks in English as a foreign language. For this purpose, reading and oral comprehension were performed along with written and oral production tasks by adults with dyslexia and their control peers. The results suggest that Spanish adults with dyslexia need more time to read English texts, and their reading comprehension is significantly worse than that of the control group. In written production, differences from the control group were found in the number of spelling errors. In addition, the oral productions showed differences in semantic errors, lexical diversity, and sentence complexity. Interestingly, the group differences for all measures were greater for the oral than for the written production tasks, leading to the hypothesis that this population is negatively affected by the spontaneity of the situation. In terms of practical implications, it seems important to provide special support for adults with dyslexia not only for written language learning but also for oral language learning in English.

Mastering more than one language is often an advantage and sometimes a necessity for living and working in a globalized world (Chávez-Zambano et al., 2017). English is considered a lingua franca and is therefore the most studied foreign language in the world. However, learning English as a foreign language (EFL)^1^ is always challenging, and it can become an especially uphill battle for people with developmental dyslexia. Despite this, there are not many studies looking at the development of EFL domains in people with dyslexia, let alone in adults (Álvarez-Cañizo et al., 2023; Helland & Kaasa, 2005).

Developmental dyslexia is characterized by problems with accurate and fluent recognition of written words and spelling (Carroll et al., 2024). In contrast to acquired dyslexia, which occurs as a result of brain injury in individuals who previously had normal reading skills, developmental dyslexia is a neurodevelopmental disorder that affects reading ability from early childhood and persists into adulthood (Kirby et al., 2024; Swanson & Hsieh, 2009). These literacy deficits have been widely studied, but the mechanisms underlying them (primarily phonological processing) appear to affect several language domains negatively. There are some studies suggesting that people with dyslexia have difficulties not only in word reading and spelling but also in reading and oral comprehension, writing composition, and speaking skills in diverse language measurements (e.g., different kinds of errors, types, tokens, lexical diversity, productivity, or mean length of utterances) (Reis et al., 2020; Snowling & Melby-Lervåg, 2016; Sumner et al., 2016; Wiseheart & Altmann, 2018), but it could depend on age.

## Language domains and dyslexia

### Reading comprehension

Initially, reading comprehension problems were considered to be a secondary effect or consequence of reading accuracy deficits in people with dyslexia (Georgiou et al., 2022). Decoding processes are thought to pose a high cognitive load for them, reducing the cognitive resources available for reading comprehension processes. However, by adulthood, decoding should be automated, making reading comprehension more dependent on other variables such as oral comprehension, general knowledge, and vocabulary competence (Brèthes et al., 2022; Ransby & Swanson, 2003).

Regarding sentence reading comprehension (time and accuracy), differences have been found between adults with and without dyslexia (Wiseheart et al., 2009). However, when working memory, word reading accuracy, and word reading speed were controlled for, these differences disappeared. For text comprehension, a meta-analysis showed that deficits are present in adults with dyslexia, but to a lesser extent than for other literacy skills (Reis et al., 2020). It should be noted, however, that different investigations produce very different results. Some studies have reported that the difficulties found in children with dyslexia are less pronounced or even disappear in adults with dyslexia (Brèthes et al., 2022; Cavalli et al., 2019; Hebert et al., 2018; Moojen et al., 2020). Conversely, adults with dyslexia have also been found to perform worse on reading comprehension questions than their control peers (Parrila & Georgiou, 2007; Ransby & Swanson, 2003). This seems to happen when a time limit is set for the task. Therefore, we can assume that compensation for core deficits is not all or nothing and depends on the presence of specific conditions such as a long and successful educational experience (Hebert et al., 2018) and, more importantly, the time constraints of the tasks (Parrila & Georgiou, 2007).

### Oral comprehension

Better performance in oral than in reading comprehension could be expected, although possible difficulties in vocabulary acquisition and verbal memory deficits might also affect oral comprehension. Accordingly, the literature reveals very heterogeneous results on the receptive vocabulary skills of adults with dyslexia. Some authors found no difference between participants with and without dyslexia (Wiseheart & Altmann, 2018; Wiseheart et al., 2009), while others show that adults with dyslexia even outperform controls on vocabulary depth measured by a definition task that assesses the accuracy and precision of word knowledge (Cavalli et al., 2016). In contrast, other studies (Callens et al., 2012; Ransby & Swanson, 2003) show weaknesses in the receptive vocabulary of adults with dyslexia. A recent meta-analysis (Reis et al., 2020) appears to confirm that adults with dyslexia have poorer general vocabulary skills than their control peers (although the effect sizes were smaller than for other reading, writing, and reading-related skills). However, this meta-analysis does not distinguish between vocabulary comprehension and vocabulary production. Thus, the debate about vocabulary comprehension skills in this population continues to range on.

In terms of the role of phonological short-term memory in oral comprehension, children with dyslexia appear to be compromised. Using a demanding sentence-picture matching task in which syntactic difficulty was manipulated, children with dyslexia were found to have low accuracy in sentence comprehension (Robertson & Joanisse, 2010). For adults, the results have been inconclusive and appear to be task-dependent. When the task consisted of short audio fragments, no differences were found between adults with and without dyslexia (Callens et al., 2012). However, when the presented passages were of increasing difficulty, adults with dyslexia scored similarly to younger adults with the same reading level and lower than adults of the same chronological age (Ransby & Swanson, 2003).

### Written production

Moreover, a growing body of evidence suggests that spelling difficulties have a detrimental effect on other aspects of the writing process, including planning, translation, and transcription (Afonso et al., 2022; Sumner et al., 2016). In this sense, the overall text quality of the written productions of children with dyslexia has been found to be poorer (Afonso et al., 2022; Sumner et al., 2016), with lower lexical diversity (Wengelin, 2007) and fewer ideas (Sumner et al., 2016) than in control peers, despite no differences being found when comparing oral compositions (Sumner et al., 2016). Furthermore, the handwriting speed of children with dyslexia is low (Afonso et al., 2020, 2022; Suárez-Coalla et al., 2020a; Sumner et al., 2013, 2014). Interestingly, in studies conducted with adults, although spelling errors persist (Li & Hamel, 2003; MacKay et al., 2019; Reis et al., 2020; Sumner & Connelly, 2020) and represent their greatest handicap (Sehlström et al., 2022), these do not seem to affect higher order skills such as quality of the ideas and organization (Connelly et al., 2006), the text’s grammar, punctuation, length, and linguistic richness (Bogdanowicz et al., 2014; Sehlström et al., 2022), or handwriting speed (Sumner & Connelly, 2020). However, these authors mention that their results could be explained by an undemanding task (a short and simple task without time pressure). Indeed, when a time-limited task was used, low legibility, grammar, and vocabulary were found in the written compositions of adults with dyslexia (MacKay et al., 2019).

### Oral production

Finally, dyslexia has often been associated with a delay in the development of oral production skills (Price et al., 2022) and lower scores in phonological recovery (Maurer et al., 2021; Suárez-Coalla et al., 2013) and sentence construction (Helland & Kaasa, 2005). In a study where adults were asked to construct sentences, the group with dyslexia was found to be significantly slower to respond to the task and to produce imprecise sentences that were poorer in terms of grammaticality and completeness (Wiseheart & Altmann, 2018).

In short, several studies have reported that adults with dyslexia have some difficulty with sentence reading comprehension, oral comprehension, spelling, and oral sentence production. However, these results are inconclusive because they are often task-dependent, with highly demanding or time-limited tasks producing the greatest differences between adults with and without dyslexia. Therefore, the present study aims to clarify the extent to which these difficulties are manifested in adults with dyslexia.

## EFL among adults with dyslexia

The ability to communicate in more than one language is known to have great benefits for people with dyslexia, both for second language learners (Kormos & Margaret, 2023) and particularly in a bilingual context (Reina et al., 2023). However, despite the advantages, learning English can still be particularly challenging for this population.

Several theories have attempted to explain the relationship between native language (L1) acquisition and second language (L2) learning. The linguistic coding differences hypothesis (LCDH) (Sparks et al., 1999, 2012) posits a directly proportional relationship between the L1 and L2 acquisition skills—in other words, phonological, orthographic, syntactic, and semantic proficiency in L1 provides the basis for the effective learning of L2. In this sense, if people show difficulties in some native language domains, it is to be expected that they will have similar difficulties in a foreign language, and even more so if the languages (native and foreign) are significantly different, causing some interference. Regarding Spanish and English languages, English has some phonemes that are not part of the Spanish phonological system (/v, ð, z, ʃ, ʒ, dʒ, h, ŋ, ɾ/) (Estebas-Villaplana, 2019), which may be difficult for some EFL learners to distinguish and produce. English syntax may also be challenging for Spanish English learners, mainly because of the complexity of English pronoun use and because the flexible word order (SVO, SOV, VSO) of the Spanish language contrasts with the subject-verb-object (SVO) structure that predominates in English (Martínez-Vázquez, 1996).

Focusing on literacy, the central processing hypothesis (CPH) (Geva & Siegel, 2000) suggests that the basic reading skills in all languages are primarily influenced by underlying cognitive factors: that is, difficulties in acquiring literacy in L1 and L2 have a common cognitive origin. These difficulties can be compounded when the languages are as different as Spanish and English. Spanish has a highly transparent orthography with a high correspondence between graphemes and phonemes (Seymour et al., 2003). In contrast, English orthography is more irregular than Spanish orthography. There are 26 letters in the alphabet but more than 40 different sounds, and some words have the same pronunciation but different spelling (e.g., “know” and “no”), while others have the same spelling but different pronunciation (e.g., “read,” infinitive and past tense) (Marks, 2007). In this regard, the script-dependent hypothesis (Geva & Siegel, 2000) postulates that in a population learning more than one language at a time, word recognition skills develop more slowly in a language with irregular orthography than in that with a regular orthography. Therefore, in these cases, in addition to the difficulties due to the interaction of the two languages (Hevia-Tuero et al., 2022), the deep orthography of languages like English may exacerbate reading problems (Reis et al., 2020). In fact, literacy problems in EFL have been found even in the high-risk non-dyslexic group (children of parents with dyslexia but without reading difficulties in the native language) (Van Setten et al., 2017).

There are few studies on the different domains of EFL in people with dyslexia, especially in adults (Sehlström et al., 2022). However, based on these theories, it can be assumed that all the difficulties described above for adults with dyslexia (pronounced in literacy, but also appearing in oral language domains) will have a strong impact on EFL learning (Helland & Kaasa, 2005; Ho & Fong, 2005; Maurer et al., 2021; Paradis, 2011).

### Reading comprehension in EFL

Studies analyzing EFL word reading skills of children with dyslexia have revealed their difficulties regardless of their native language (Polish: Łockiewicz & Jaskulska, 2016; Chinese: Chung & Ho, 2010; Italian: Fazio et al., 2021). Consistently, Spanish-speaking children with dyslexia have shown problems using English grapheme-phoneme rules, forcing them to adopt a lexical strategy to read English words (Suárez-Coalla et al., 2020b). Similar results were found in adults, concluding not only that people with dyslexia were slower and less accurate in reading words than controls but also that response latencies were more pronounced in EFL than in L1 (Oren & Breznitz, 2005). In any case, it is well known that reading a text is much more than decoding individual words. To really understand a text requires different skills, including knowing grammar and vocabulary, examining text structure, making inferences, and self-monitoring the reading process (Ouellette & Beers, 2010; Suggate et al., 2018).

### Oral comprehension in EFL

In addition, children with dyslexia show poorer performance than their peers in various processes related to EFL oral comprehension: receptive vocabulary (Geva & Massey-Garrison, 2013; Ho & Fong, 2005; Łockiewicz & Jaskulska, 2016), morphology (Helland & Kaasa, 2005), and syntax (Geva & Massey-Garrison, 2013). Therefore, it is not surprising that children with dyslexia performed worse than controls when answering questions about paragraphs presented orally (Geva & Massey-Garrison, 2013).

### Writing production in EFL

Moving into writing skills, spelling may represent the most challenging area of EFL for both children (Helland & Kaasa, 2005; Palladino et al., 2016) and adults (Lindgrén & Laine, 2011) with dyslexia. This is due to a mixture of individual and linguistic reasons. On the one hand, the neurobiological characteristics of people with dyslexia lead them to develop weak orthographic representations of the words (Łockiewicz & Jaskulska, 2016; Suárez-Coalla et al., 2020a) that result in an erroneous “spelling by ear” (Andreou & Baseki, 2012). On the other hand, it has already been mentioned that the orthographic inconsistency of languages such as English has a negative impact on dyslexic symptoms (Caravolas et al., 2013; Landerl et al., 2013; Reis et al., 2020; Seymour et al., 2003; Ziegler et al., 2010). In this line, in contrast to children whose spelling difficulties negatively affect other aspects of their written productions (Afonso et al., 2022), when upper-secondary students with dyslexia are asked to write in their native language, their difficulties are reduced to spelling. However, when the same population writes in EFL, limitations also appear in text cohesion, vocabulary, and punctuation measures (Sehlström et al., 2022).

### Oral production in EFL

As far as oral production is concerned, studies with children have shown that poor readers have lower scores in productive vocabulary (Maurer et al., 2021) and sentence construction (Helland & Kaasa, 2005) in EFL. This might be expected given that people with dyslexia show a working memory deficit (Beneventi et al., 2010) and that speaking is known to be a complex task that requires large cognitive resources, even more so when speaking in a non-native language. There is growing evidence linking verbal working memory to the acquisition of vocabulary and grammar in the native language (Adams & Gathercole, 1996, 2000), but especially in explicit L2 learning by both children (French & O’Brien, 2008; Masoura & Gathercole, 2005) and adults (Martin & Ellis, 2012; Tagarelli et al., 2011; Williams & Lovatt, 2003). In this regard, a study comparing native Dutch speakers and Dutch learners showed that phonological memory and grammar were weakly correlated in L1 and strongly correlated in L2 learners (Verhagen et al., 2015).

Considering the above, it can be stated that children with dyslexia show difficulties in a wide range of EFL domains. However, much less research has been done on the adult population. Specifically, we still know little about the EFL reading, oral comprehension, and oral production skills of adults with dyslexia. Furthermore, previous research has tended to focus on individual language skills in isolation, with few studies comparing multiple language skills. This highlights the need for a comprehensive understanding of the overall language skills of adults with dyslexia.

## The present study

The present study aims to investigate how native Spanish-speaking adults with developmental dyslexia perform different language tasks in EFL. This will give us a more complete picture of the strengths and weaknesses of this population when it comes to EFL learning. Specifically, we seek to address the following issues:

- Are there differences in the EFL skills of adults with and without dyslexia?
- More specifically, do adults with dyslexia differ from the controls in oral and reading comprehension in EFL? Are there differences in the number of correct answers or in the time taken to complete tasks?
- Do adults with dyslexia differ from the controls in oral and written production? Are the differences in the types of error, in the richness of productions, or in the productivity?

For this purpose, reading and oral comprehension, and written and oral production tasks were performed by adults with dyslexia and their control peers. According to literature, it is expected that adults with dyslexia will show poorer performance in all tasks, especially those involving written language. Regarding the research question on reading and oral comprehension, it is hypothesized that the DYS group will need more time to complete both tasks. However, statistical differences in the number of correct answers are only expected in the reading comprehension task, as previous studies have shown difficulties for adults in word reading (Oren & Breznitz, 2005), but not in similar short audio fragment listening tasks (Callens et al., 2012). Moving on to writing and oral production, adults with dyslexia are supposed to be less competent because these are two complex tasks that, when performed in EFL, are particularly demanding on working memory. We also hypothesized that they would have many spelling errors due to their core deficit (Helland & Kaasa, 2005; Palladino et al., 2016).

## Materials and methods

### Participants

A total of 36 young adults (26 girls and 10 boys) aged between 18 and 25 years (*M* = 20 years, 1 month, *SD* = 1.70) participated in the study. Half of the participants had a diagnosis of dyslexia (DYS), and half were typical readers (CON). The two groups were matched for age, gender, educational level, and number of years studying English (see Table 1). All the participants were native Spanish speakers who had learnt English as a foreign language. They did not speak any other languages and had no known motor, cognitive, or perceptual disorders.

**Table 1:** Participants’ demographic information in the DYS and CON groups

	CON (*n* = 18)	DYS (*n* = 18)
Age		
Mean (SD)	20 years, 2 months (1.70)	19 years, 11 months (1.73)
Range	18–23	18–23
Gender	Female: 11 Male: 7	Female: 11 Male: 7
English level	Basic: 12 B1: 3 B2: 3	Basic: 12 B1: 3 B2: 3
Level of education being pursued	Basic professional training: 3 Advanced professional training: 1 University studies: 14	Basic professional training: 3 Advanced professional training: 1 University studies: 14

Basic: basic knowledge of English (they had studied it as a compulsory subject at school between the ages of 5 and 18). B1 qualification: intermediate level, Preliminary English Test in Cambridge measures. B2 qualification: advanced level, Cambridge First Certificate

Participants with dyslexia were recruited from the Language Psychology Laboratory at the University of Oviedo using a deliberate sampling method. Eleven of the adults had been diagnosed with dyslexia in childhood and were experiencing persistent difficulties with reading and writing. Seven of the participants were evaluated as having suspected literacy difficulties and a history of poor academic performance. In these cases, they were assessed using PROLEXIA (Cuetos et al., 2020), a test with normative data to aid in the differential diagnosis of dyslexia. Since the scores indicated a high or very high risk of dyslexia, and the subjective assessment of the specialist pointed in the same direction, the subjects were considered to have dyslexia and were included in the DYS group. Participants in the control group were recruited from universities and vocational training centers in Spain. Both the participants with dyslexia and the controls were volunteers collaborating with the Language Psychology Laboratory of the University of Oviedo. It was difficult to find participants who fit the specific profile we wanted to analyze. Therefore, 16 participants (eight with and eight without dyslexia) were excluded from the study because they had studied outside the Spanish education system, because they were not between the ages of 18 and 25, or because they were multilingual.

All participants were attending post-compulsory studies (28 were high school students, two were in higher vocational education, and six were in basic vocational education). In terms of their English level, 20 of the participants (ten DYS and ten CON) had a basic knowledge of English (they had studied it as a compulsory subject at school between the ages of 5 and 18 for approximately 4 h per week). The rest had continued to learn English after leaving school, six (three DYS and three CON) achieving a B1 qualification (intermediate level, Preliminary English Test in Cambridge measures), and six (3 DYS and 3 CON) achieving B2 (advanced level, Cambridge First Certificate).

To validate the allocation of participants to the different groups, a reading task in Spanish (L1) was administered. Participants had to read aloud two lists of 28 words and pseudowords of varying length and lexical frequency, selected from the EsPal database (Duchon et al., 2013) (see Appendices 1 and 2). Reading accuracy and speed were recorded. From the data, we could prove that adults with dyslexia performed significantly below the control group both in accuracy and in word and pseudoword reading speed. See Table 2.

**Table 2:** Reading scores of the adult participants (means and standard deviations)

	Control (*n* = 18) *M* (*SD*)	Dyslexia (*n* = 18) *M* (*SD*)	*p*-value*
**Words**			
Accuracy	27.83 (0.38)	26.67 (1.19)	< 0.001
Speed (s)	15.83 (4.82)	24.33 (5.61)	< 0.001
**Pseudowords**			
Accuracy	26.22 (1.23)	23.83 (1.54)	< 0.001
Speed (s)	25.94 (9.02)	37.72 (7.69)	< 0.001

*Holm-Bonferroni corrected *p*-value

## Materials

Four English tasks were created to assess the different language domains: reading and oral comprehension, writing, and oral production. The task development was supervised by two English teachers from a secondary school.

### Reading comprehension

The reading comprehension task consisted of reading two independent texts and answering comprehension questions. These texts were adaptations of the EBAU English language exams (the official exam to enter university in Spain). The adjustments consisted of reducing the number of words in the text and the questions. In order to verify that the two texts were of the same level, the online Readability Test Tool (Readability Test, 2024) was used. The final texts consisted of 199 words (Text 1) and 186 words (Text 2), focusing on the topics of air travel and smartphone dependency, respectively. Each reading had a time limit of 7 min, enabling 34 out of the 36 participants to complete both tasks successfully. The assessment was conducted online (see the “Procedure” section). The following measures were considered: number of correct answers, number of times the text was consulted, reading time, and total task time. See Table 3.

**Table 3:** Measures considered in each of the language tasks

**Reading comprehension**	
Number of correct answers	Participants were asked to answer four open-ended questions (two for each reading) by reformulating the information in the text—that is, without copying it verbatim. Each question was scored between 0 and 2: 0 if the answer was incorrect, 1 if the answer was correct but not reformulated, and 2 if the answer was correct and reformulated. Participants were warned that spelling errors would not be taken into account. The maximum score was 8 points
Number of times the text was consulted	The screen recordings made it possible to note how often the participants scrolled up to consult the text again while answering the questions
Reading time	Time (in seconds) needed to read the texts. The participant’s screen was recorded to calculate the time spent viewing the text (see the Procedure section)
Total task time	Total time (in seconds) taken to read the text and answer the questions
**Oral comprehension**	
Number of correct answers	One point for each correct answer. The maximum score possible was 8 points
Number of times the audio was consulted	Number of times the audio files were listened to (noted by the researcher)
Total task time	Total time (in seconds) taken to complete the task (reading the questions, listening to the audios, and answering the questions)
**Written production**	
Percentage of errors	The number of errors in relation to the number of written words (see Table 10 in the Appendix). Initially, five major blocks of errors (spelling, grammatical, semantic, punctuation, and self-correction) were considered, which included different sub-types of error. The percentage of each subtype within the different categories was calculated. The different types of spelling error are letter substitution, letter omission/addition, missing/unnecessary space between words, letter inversion, errors resulting from a clear “spelling by ear,” and incorrect use of upper/lower case letters. Grammatical errors include incorrect verb tense, misuse of the -s in the third person singular, incorrect singular/plural agreement, incorrect word order, missing/unnecessary grammatical word, substitution of a grammatical word, incorrect derivation of words, and incorrect use of’s in possessives. Semantic errors are substitution of a content word, invention of a word, and missing/unnecessary content word
Mean length of utterances in words (MLU)	A measure of syntactic complexity calculated by dividing the number of words by the number of utterances
Subordination index (SI)	A measure of syntactic complexity that gives the ratio of the total number of clauses to the total number of c-units (independent clauses with their modifiers) (Miller & Iglesias, 2012)
Lexical diversity	Guiraud index of lexical diversity (type/√token) was used to know how many different words appear in a text, controlling for differences in the length of the narratives
Writing productivity	Number of words written per minute
Word types	Percentage of determiners, adjectives, nouns, personal pronouns, other pronouns, auxiliary modals, auxiliary operators, verbs, copula forms, verb particles, adverbs, intensifiers, prepositions, existential there, coordinators, subordinators, infinitives, and negation words
**Oral production**	
Percentage of errors	Pronunciation errors were added to grammatical and semantic errors (see Table 10 in the Appendix)
Mean length of utterances in words (MLU)	See written production
Subordination index (SI)	See written production
Lexical diversity	See written production
Oral productivity	Number of words spoken per minute
Word types	See written production
Percentage of mazes	Series of words or unattached fragments which do not contribute to the meaning in the ongoing flow of language
Number of interjections per minute	Any kind of filler with no linguistic content was considered an interjection
Pauses	The number of pauses per minute, the average pause duration, and the percentage of pause time (silence time in relation to total speech duration) were measured. Any silence longer than 100 ms was considered a pause. Praat software was used to perform these analyses (Boersma & Weenink, 2024)

### Oral comprehension

A free access sample of the Cambridge Preliminary English Test (PET) was used to assess oral comprehension. Participants listened to four audios of 1 min each representative of the B1 level. They could listen to them as many times as they wanted. After listening to each audio, they had to read and answer two test questions with three alternatives (only one was correct). No time constraints were imposed for reading and answering the questions. The following measures were considered: number of correct answers, number of times consulting the audios, and total task time. See Table 3.

### Written production

In the written production task, participants were given a maximum of 7 min to create a written composition on one of two proposed themes: “Your worst travel experience” or “Healthy lifestyle.” These two topics were inspired by the official English language exams. The minimum required length was six lines. The compositions were transcribed and coded by SALT software (Miller & Iglesias, 2012). The following measures were considered: percentage of errors, mean length of utterances in words (MLU), subordination index (SI), lexical diversity, writing productivity, and word types. See Table 3.

### Oral production

**Fig. 1:**
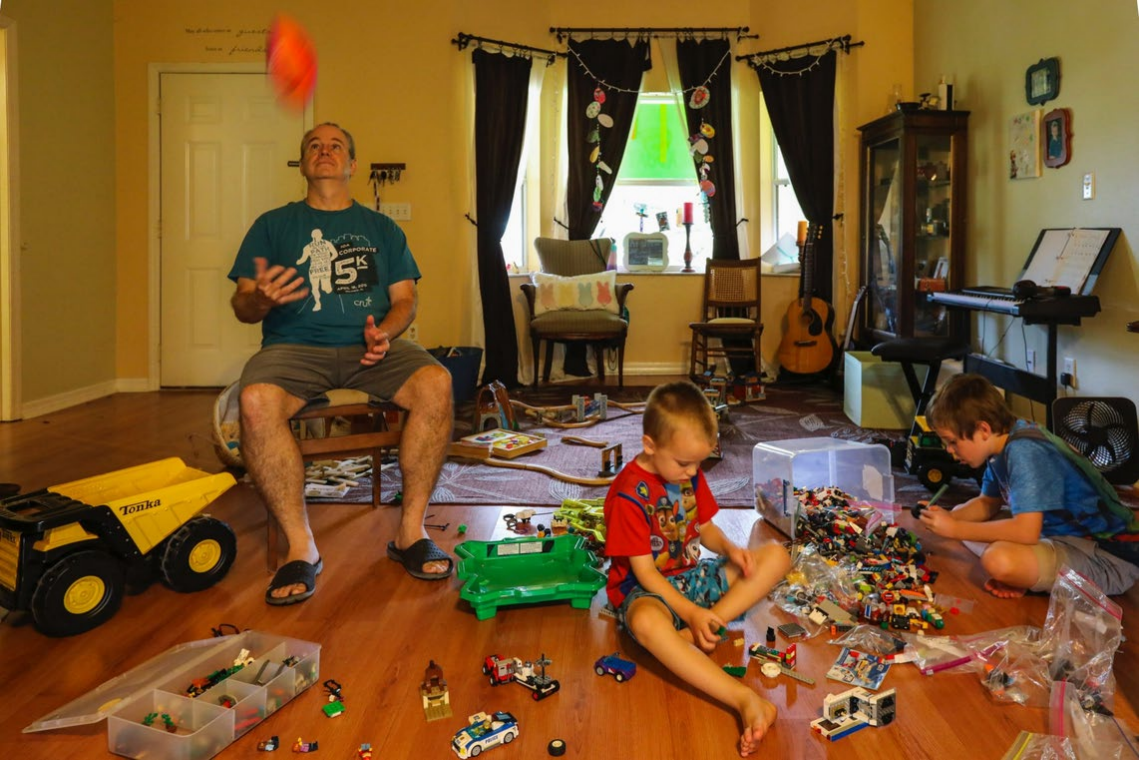
Picture to be described in the oral production task

The oral production task consisted of an oral composition task based on a picture (see Fig. 1). The maximum time available was 2 min, but participants could finish earlier if they ran out of things to say. Responses were recorded, transcribed by a bilingual university student, and coded using SALT software (Miller & Iglesias, 2012). The following measures were considered: percentage of errors, mean length of utterances in words (MLU), subordination index (SI), lexical diversity, speaking productivity, word types, percentage of mazes, and number of interjections per minute. See Table 3.

## Procedure

The research design was approved by the Research Ethics Committee of the University of Oviedo. In accordance with the principles of the Declaration of Helsinki and the Spanish Personal Data Protection Act (15/1999 and 3/2018), before starting the experimental tasks, all participants received relevant information about the purpose of the study, the nature of the tasks, and their duration. Written informed consent was obtained from the participants.

Participants enrolled via a Google Forms questionnaire, which was used to assess whether they met all the eligibility criteria. After that, they were scheduled to meet with the researcher. The experimental sessions were conducted online, with the participant and the researcher being connected at the same time. All tests were monitored using the Zoom platform and participants’ responses were stored live using Google Forms and an ad hoc web page. During the session, both the participant’s screen and the image from their camera were recorded using the *iMovie* (*iMovie*, 2023) program for further review and data collection. The four tasks lasted approximately 80 min.

## Analysis

Data were analyzed using JASP software version 0.18.3 (JASP Team, 2024). First, the Shapiro–Wilk normality test was applied: since most of the results were above 0.05, it was decided to apply parametric statistics. An independent samples *t*-test was used to compare performance between the groups with and without dyslexia. For each variable, a *p-*value was calculated to analyze whether the differences between groups were statistically significant, and Cohen’s *d* was used to see the size of these differences. Since multiple independent sample *t*-tests were performed within each language domain,* p*-values were adjusted using the Horn-Bonferroni method to control for the risk of Type I errors. This involves ordering the *p*-values from smallest to largest and then adjusting the significance threshold for each test. In this way, we were able to increase our statistical power while maintaining control over the family-wise error rate.

## Results

### Reading comprehension

We found a group effect in correct answers, the number of times the text was consulted, and reading time. The DYS group answered fewer questions correctly than the CON group even though they consulted the text more often and they spent considerably more time reading the text. However, no differences were found in the total time spent on the task (both reading the text and answering the questions) (see Table 4).

**Table 4:** Reading measurements in CON and DYS groups

	Control (*n* = 18) *M* (*SD*)	Dyslexia (*n* = 18) *M* (*SD*)	*p-*value*	*d* value
Number of correct answers (out of 8)	6.11 (1.81)	4.17 (2.15)	0.012	0.978
Number of times the text was consulted	9.33 (4.02)	15.33 (5.58)	< 0.001	− 1.234
Reading time (s)	252.67 (97.99)	389.28 (116.91)	< 0.001	− 1.267
Total task time (s)	613.61 (198.73)	668.33 (110.72)	0.315	− 0.340

*Holm-Bonferroni corrected *p*-value

### Oral comprehension

No significant differences were found between the DYS and CON groups in any of the measures analyzed: number of correct answers, number of times listening to the audios, and total task time. However, the results were slightly lower in the DYS group on all three measures (see Table 5).

**Table 5:** Oral comprehension measurements in CON and DYS groups

	Control (*n* = 18) *M* (*SD*)	Dyslexia (*n* = 18) *M* (*SD*)	*p*-value*	*d* value
Number of correct answers (out of 8)	6.61 (1.15)	5.94 (1.31)	0.336	0.543
Number of times the audio was consulted	9.28 (2.16)	10.89 (3.71)	0.242	− 0.531
Total task time (s)	416.11 (142.15)	444.89 (199.63)	0.634	− 0.160

*Holm-Bonferroni corrected *p*-value

### Written production

After the analyses, we only found a group effect in the number of spelling errors (*p* =  < 0.001; *d* =  − 1.305), as the DYS group made more spelling errors than the CON group (see Table 6). However, we did not find a group effect for the remaining error types (grammatical, semantic, punctuation, and self-correction), in the subtypes of errors, in the richness of productions, or in the use of different word types.

**Table 6:** Comparisons of different types of errors and richness of productions in writing production task between CON and DYS groups

	Control (*n* = 18) *M* (*SD*)	Dyslexia (*n* = 18) *M* (*SD*)	*p*-value*	*d* value
Errors				
% Spelling errors	2.59 (2.03)	8.51 (6.09)	< 0.001	− 1.305
% Grammatical errors	6.79 (5.27)	8.92 (7.06)	> 1.00	− 0.343
% Semantic errors	2.45 (2.48)	2.71 (2.52)	> 1.00	− 0.104
% Punctuation errors	1.61 (2.28)	1.57 (2.11)	0.958	0.018
% Self-corrections	3.66 (2.85)	3.90 (3.50)	> 1.00	− 0.235
Richness of the productions				
MLU	17.25 (5.47)	15.17 (5.52)	0.262	0.389
SI	2.41 (0.64)	2.05 (0.78)	> 1.00	0.514
Lexical diversity	5.97 (0.63)	5.44 (0.71)	> 1.00	0.799
Writing productivity	17.15 (6.96)	12.55 (3.27)	0.128	0.845

*MLU* mean length of utterances in words, *SI* subordination index *Holm-Bonferroni corrected *p*-value

### Oral production

In the oral production from a picture, we found a group effect for semantic errors, as the DYS group committed more errors than the CON group. The difference was also significant for the semantic sub-error of substituting content words (*M*_dys_ = 55.56, *SD* = 38.35; *M*_con_ = 5.56, *SD* = 23.57, *p* = 0.000, *d* =  − 1.571). However, no differences were found for pronunciation errors or self-corrections, in the use of different word types, in the percentage of mazes, or in the number of interjections per minute. Regarding the richness of the productions, we found a group effect for SI and lexical diversity (see Table 7).

**Table 7:** Comparisons of different types of errors and richness of productions in oral production task between CON and DYS groups

	Control (*n* = 18) *M* (*SD*)	Dyslexia (*n* = 18) *M* (*SD*)		*p-*value*	*d* value
Errors					
% Grammatical errors	9.05 (4.29)	13.79 (7.85)	0.155		− 0.749
% Semantic errors	0.05 (0.22)	3.04 (2.56)	< 0.001		− 1.649
% Pronunciation errors	0.69 (1.49)	1.74 (3.06)	0.591		− 0.439
% Self-corrections	0.80 (1.10)	0.96 (1.43)	0.712		− 0.124
Richness of the productions					
MLU	14.68 (4.12)	11.88 (3.80)	0.168		0.705
SI	1.91 (0.53)	1.43 (0.51)	0.048		0.940
Lexical diversity	5.50 (0.69)	4.67 (0.86)	0.021		1.063
Oral productivity	79.54 (29.10)	68.41 (22.43)	0.416		0.428

*MLU* mean length of utterances in words, *SI* subordination index *Holm-Bonferroni corrected *p*-value

### Comparison of written and oral productions

Finally, when comparing the performance on oral and written production tasks, larger group differences were found for oral production tasks for all measures, both in the analysis of errors and the richness of the productions (see Tables 6 and 7).

## Discussion

The aim of this study was to assess the specific difficulties of Spanish adults (ages 18–25) with dyslexia when using EFL. To this end, participants with dyslexia and their control peers were faced with reading and oral comprehension, writing, and oral production tasks. The levels of competence and the types of error made by each group were examined.

### Reading comprehension

The results suggest that Spanish adults with dyslexia need more time to read English texts and yet their reading comprehension is significantly worse than that of controls. The scientific literature seems to agree that adults with dyslexia, despite their weakness in word reading, tend to compensate for their difficulties in simple L1 text comprehension tasks and perform them similarly to their peers without dyslexia (Brèthes et al., 2022; Cavalli et al., 2019; Moojen et al., 2020; Reis et al., 2020). However, differences appear when the difficulty is increased, for example, by time constraints (Parrila & Georgiou, 2007; Wiseheart et al., 2009). Our results in EFL reading comprehension show that although participants with dyslexia could spend as much time as they wanted on the task, their scores were still worse than those of controls. This can be interpreted to mean that the use of L2 also increases the difficulty of the tasks, making their competence lower than expected.

It should be noted that the DYS group took longer to read the passages, but the total time to complete the task did not differ between the groups. This suggests to us that the participants with dyslexia took less time to answer the questions, which could explain their poorer performance. Perhaps the DYS group tended to respond quickly to compensate for the extra time they spent reading the text. It may be that they have internalized this strategy to such an extent that they use it even when they know they have no time limit to complete the task. Given the interest in these findings and their possible practical implications, this line of research should be further explored.

### Oral comprehension

The oral comprehension task was the only one in which there were no differences between adults with and without dyslexia in any of the areas assessed (number of correct answers, time to complete the task, and number of times the audio was heard). However, the DYS group scored slightly lower on the three tasks (although the differences were not significant). These data are in line with previous studies reporting a quite consistent deficit in adults with dyslexia that makes their average performance lower on almost all tests (Callens et al., 2012; Hatcher et al., 2002). However, these results can also be interpreted as a consequence of the methodology used to assess oral comprehension. Participants had to read the questions which, although being simple, may have influenced the accuracy of the answers and the time taken to complete the task. This raises the question of how tests in educational contexts should be adapted to meet the needs of people with these difficulties.

No studies were found that compared the oral comprehension skills of adults with dyslexia in foreign language. In the child population, and contrary to the findings of the present study, CON children performed better than DYS children in answering oral comprehension questions in EFL (Geva & Massey-Garrison, 2013). It is difficult to hypothesize the reason for these differences. On the one hand, we might think that oral comprehension difficulties disappear with age. However, it could also be that in our research, participants could listen to the audio as many times as they wanted, whereas in the previously cited report, participants could only listen to the audio once, which could influence the results. Either way, further research would be needed to clarify this issue. Other studies on adults with dyslexia, but in their L1, show that they perform poorly on complex sentences (Callens et al., 2012; Wiseheart et al., 2009) and passage (Ransby & Swanson, 2003) oral comprehension tasks. These data seem to suggest that adults with dyslexia may have difficulties with oral comprehension, but our results did not go in this direction. Future studies in which the input is heard only once will need to shed light on this aspect.

### Written production

With regard to the writing composition, the DYS group made significantly more orthographic errors than the control group. However, there were no differences in terms of grammatical, semantic, and punctuation errors. In accordance with the initial hypothesis, spelling errors are also characteristic of people with dyslexia in EFL. Moreover, it is known that the orthographic inconsistency of languages such as English exacerbates these difficulties (Caravolas et al., 2013; Landerl et al., 2013; Reis et al., 2020; Seymour et al., 2003; Ziegler et al., 2010). Previous studies comparing the spelling of DYS and CON children in EFL picture-elicited narratives (Andreou & Baseki, 2012) show a tendency of children with dyslexia to “spell by ear,” a type of error that, according to our results, practically disappears in adults. It could be assumed that adults with dyslexia are aware of the differences between Spanish and English spellings and avoid this phonological writing. Nevertheless, they continue to produce many spelling errors because of the weak orthographic representations of the words (Suárez-Coalla et al., 2020b).

Focusing on the richness of written productions, previous literature shows that while adults with dyslexia who perform L1 writing tasks show only poor spelling, when the same group performs writing tasks in EFL, differences from the CON group also emerge in cohesion, language use, and punctuation (Sehlström et al., 2022). Conversely, the present study found no group differences in measures of grammatical complexity (MLU or SI), nor in the richness of the vocabulary used (lexical diversity). In any case, the DYS group shows weaker scores in these skills, so we cannot rule out that the lack of significant differences is due to the small sample size (*n* = 18). In this line, Sehlström et al. (2022) posit that the differences in lexical diversity are partly explained by their poor spelling, which may have a negative impact on their overall text quality. However, the present study found that when adults with dyslexia produce oral narratives, they also show less lexical diversity than the controls. In fact, their lexical diversity impairments are greater in oral than in written tasks. Therefore, it is suggested that the vocabulary limitations in their writing are not due to an attempt to avoid words they do not know how to spell, but rather that adults with dyslexia have a less expressive vocabulary in EFL.

### Oral production

Finally, analysis of the oral production skills from a picture gives interesting results. The DYS group made more grammatical errors and produced syntactically simpler (SI) sentences. These results were expected, considering that children have shown poor sentence construction in EFL (Helland & Kaasa, 2005) and that adults with dyslexia tend to produce sentences that are poorer in grammaticality and completeness in L1 (Wiseheart & Altmann, 2018). In addition, adults with dyslexia had more semantic errors, used more content words incorrectly, and had significantly less lexical diversity than control participants, reflecting their difficulties with oral vocabulary use. This supports the idea that the difficulties with productive L2 vocabulary observed in children are maintained in adults (Maurer et al., 2021). On the contrary, no differences were found between people with and without dyslexia in verbal fluency (on measures of percentage of mazes, number of interjections per minute, and pauses), which is consistent with findings of children in L1 (Pistono et al., 2024).

### Comparison of written and oral productions

Interestingly, when we compare the writing and oral productions, we see that the group differences are greater in oral than in writing tasks in all the measures analyzed (both in the percentage of errors and in the grammatical and semantic richness of the productions). It is known that people with dyslexia have working memory deficits (Beneventi et al., 2010; Hatcher et al., 2002) and difficulties in accessing the phonological form of words (Suárez-Coalla et al., 2013). This is probably why their difficulties are accentuated in time-limited language tasks (Parrila & Georgiou, 2007), as they may need more time to plan what to say, to select the vocabulary, and to choose the grammatical structure. Therefore, it would appear logical that when speaking (where they have to speak on the fly, without much time to think), this is reflected in more errors and simpler productions in terms of grammar and vocabulary. In this case, it seems that small working memory deficits in adults with dyslexia (Hatcher et al., 2002; Wiseheart et al., 2009) that do not affect the L1 could lead to poorer performance in L2 tasks (Verhagen et al., 2015) that require spontaneity, thus explaining their poorer performance on oral tasks than on written tasks. This gives rise to our hypothesis about the existence of a foreign language spontaneity deficit in adults with dyslexia. However, it is important to acknowledge that the present study was not specifically designed to compare performance between oral and written productions, so that these results may be influenced by other factors, including the methodology used to assess the participants or other cognitive aspects not considered in this investigation. Given the potential pedagogical implications, further research is needed to explore alternative explanations for the observed results or to confirm the hypothesized existence of a foreign language spontaneity deficit.

## Conclusions

From all of the above, we can conclude that adults with dyslexia perform worse than controls in reading comprehension, written production, and oral production in EFL. Regarding the last two, the differences from the control group appeared in the spelling errors of the written productions, while in the oral tasks, the differences appeared in the semantic errors, in the lexical diversity, and in the syntactic complexity. Finally, another interesting finding is that the group differences for all measures are greater in oral tasks than in written tasks. This implies a new line of research, but it could be hypothesized that adults with dyslexia speaking in EFL are particularly disadvantaged by the spontaneity of the situation.

## Practical implications

It appears that some language difficulties that adults with dyslexia might be expected to have overcome in their native language persist in EFL. These findings need to be supported by future studies, but if they are, they would have important practical implications. Since the difficulties in L1 and EFL are different, we should consider that the way of teaching and curricular adaptations cannot be the same in both languages. For example, although replacing a written test with an oral test is probably an appropriate measure in the L1, we have observed that this might be unfavorable in the EFL, especially if we want to measure grammar or vocabulary performance. As stated in previous literature, it is also important to note that adults with dyslexia need additional time to complete print and oral tasks effectively in EFL. That is why we should avoid tasks that emphasize spontaneity or speed of response. For another example, rather than asking a student directly, it is advisable to share the questions and give them time to think about the answers, make notes if necessary, and then respond. Finally, spelling remains one of their greatest weaknesses, so we need to put special emphasis on the assistance they may need to compensate for this and not insist on the mistakes they make. In short, and despite their spelling struggles, we cannot limit ourselves to thinking that their difficulties are only in the written language, but must also provide them with support in other areas of oral language.

## Limitations

The outcomes of the present study help us better to understand how Spanish adults with dyslexia approach the management of EFL. However, it is necessary to consider some of the study’s limitations. On the one hand, it is difficult to oversee the exposure to English that the participants have had throughout their lives. Some of these variables have been controlled for, but it is well known that it is impossible to find participants with exactly the same linguistic background, and even more so in the adult population. It would also be very informative to consider L1 language performance in order to study cross-linguistic transfer. It should also be noted that the L1 of the participants was Spanish, so care should be taken when extrapolating the results to speakers of other L1s. Finally, the size of the group was smaller than we would like, so the results must be considered with caution. In any case, more studies are needed to deepen the management of different language domains in EFL by adults with dyslexia.

## Appendix 1

Table 8

**Table 8:** Length and frequency of items in word reading tasks

Word	Frequency	Length
pufo	0.01	4
cubilete	0.27	8
tuno	0.30	4
dedal	0.33	5
esquirol	0.49	8
comba	0.74	5
estornudo	0.88	9
erizo	1.62	5
diva	2.29	4
topología	3.06	9
desalojo	3.63	8
penumbra	4.06	8
opositor	5.46	8
petrolero	5.66	9
inca	11.50	4
despacho	18.15	8
ordenador	19.54	9
dedo	19.99	4
polvo	26.57	5
ética	26.67	5
aventura	27.83	8
árbol	49.10	5
tendencia	49.10	9
plano	59.11	5
propósito	64.42	9
caballero	66.79	9
toma	94.44	4
copa	117.71	4
*M* =	**24.48**	**7**

## Appendix 2

Table 9

**Table 9:** Length of items in pseudoword reading tasks

Word	Length
dita	4
edes	4
orfa	4
liña	4
puco	4
tero	4
arajo	5
corla	5
consé	5
depal	5
domel	5
erimo	5
pinda	5
posca	5
defegada	8
desatojo	8
diración	8
esquijol	8
idólapla	8
oñositor	8
apruencia	9
colemiata	9
esporsudo	9
pametería	9
porífota	8
pegrolero	9
tesjerete	9
tonolodía	9
*M* =	7

## Appendix 3

Table 10

**Table 10:** Error coding and examples

Code	Error	Example
**%SE**	Spelling errors (sum of SE:Sub, SE:Omi, SE:Addi, SE:Spa, SE:Inv, SE:Pgc and SE:Ulc)	
%SE:Sub	Letter substitution	“Lifestile” for lifestyle
%SE:Omi	Letter omission	“Diferent” for different
%SE:Addi	Letter addition	“Breaksfast” for breakfast
%SE:Spa	Missing or unnecessary space between words	“Everytime” for every time
%SE:Inv	Letter inversion	“Miskate” for mistake
%SE:Pgc	Errors resulting from a clear “spelling-by-ear” (Andreou G, Baseki J 2012)	“Cauntry” for country
%SE:Ulc	Incorrect use of upper or lower case letters	“i”—I
**%GE**	Grammatical errors (sum of GE:Time, GE:Third, GE:Num, GE:Der, GE:Ord, GE:MisW, GE:UnnW and GE:Sus)	
%GE:Time	Incorrect verb tense	“Yesterday we go to” for Yesterday we went
%GE:Third	Misuse of the -s in the 3rd person singular	“The plane start to fall” for The plane starts to fall
%GE:Num	Incorrect singular/plural agreement	“Four day per week” for Four days per week
%GE:Ord	Incorrect word order	“I and my friends” for My friends and I
%GE:MisW	Missing grammatical word	“In morning” for In the morning
%GE:UnnW	Unnecessary grammatical word	“I travelled by the plane” for I travelled by plane
%GE:Sus	Substitution of a grammatical word	“In breakfast I drink milk” for For breakfast I drink milk
%GE:Der	Incorrect derivation of words (for an incorrect use of an affix) or an incorrect use of “’s” in possessives	“Is very normally” for Is very normal
%SeE	Semantic errors (sum of SeE:Sus, SeE:Inven, SeE:MisW and SeE:UnnW)	
%SeE:Sus	Substitution of a content word	“I tent” for I try
%SeE:Inven	Invention of a word	“Supere” for overcome
%SeE:MisW	Missing content word	“Regarding eating, I would like to have more habits” for Regarding eating, I would like to have more healthy habits
%SeE:UnnW	Unnecessary content word	“I bought another new tickets” for I bought new tickets
**%PE**	Punctuation errors	“¡It was great!” for It was great!
**%SC**	Self-corrections	Strikethroughs

## Footnotes

1 At this point, we think it is important to note the difference between acquiring a second language and learning a foreign language. Acquiring a second language is an unconscious process in which a person implicitly absorbs speech samples and instinctively reproduces them. In contrast, learning a foreign language is a conscious process in which a individual explicitly learns speech samples and deliberately produces them (Turkevych, 2019). Beyond this definition, we know that there is considerable overlap in the way language learning occurs in both processes (e.g., young second language learners receive explicit language instruction, while foreign language learners can easily expose themselves to authentic linguistic input thanks to the Internet). However, it cannot be denied that the process of language learning is significantly different in the two cases. Moreover, foreign language learners have fewer opportunities for authentic language input than second language learners and may not feel the same need to learn English. Therefore, it is fair to say that the different characteristics of these populations imply that they need to be studied separately.
